# Supplementary material for: GDSL Esterase/Lipase GELP1 Involved in the Defense of Apple Leaves against Colletotrichum gloeosporioides Infection
Source: Int J Mol Sci. 2023 Jun 19;24(12):10343. doi: 10.3390/ijms241210343 (PMC10299637; doi:10.3390/ijms241210343)
Supplement: Supplementary file 1 [file ijms-24-10343-s001.zip › Figures S1-S3.pdf]

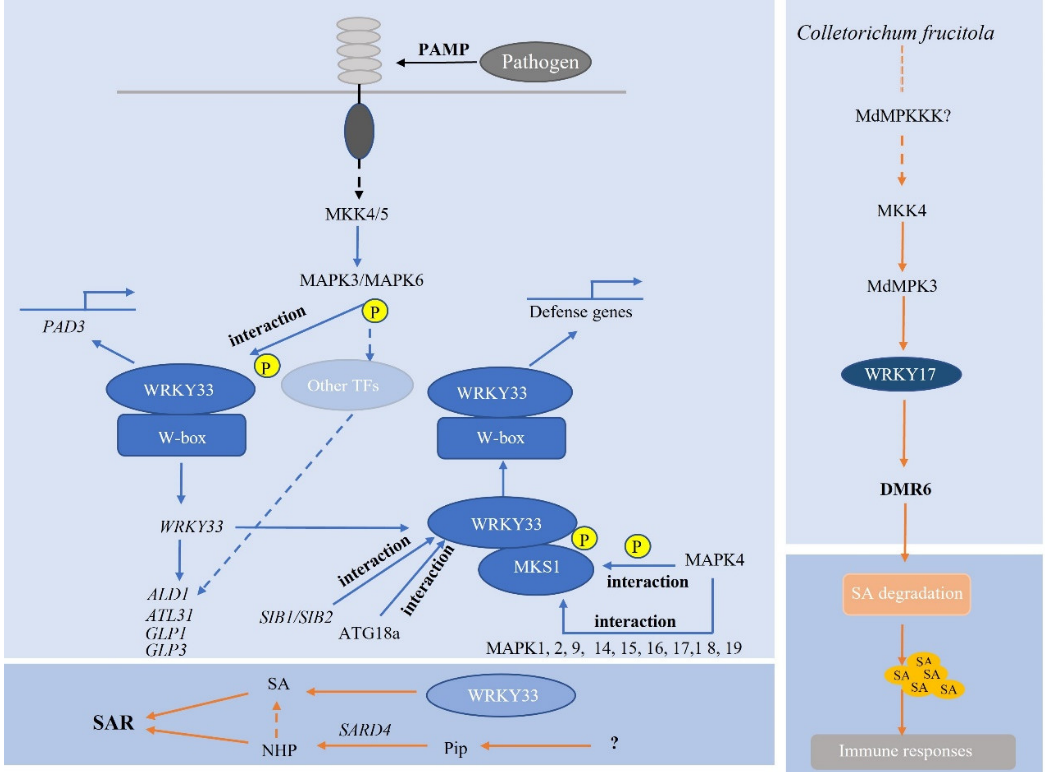

Figure S1. Schematic model of AtwrKY33 and MdWRKY17 involved in downstream pathogenic pathogens of the MAPK cascade[31, 36-42].

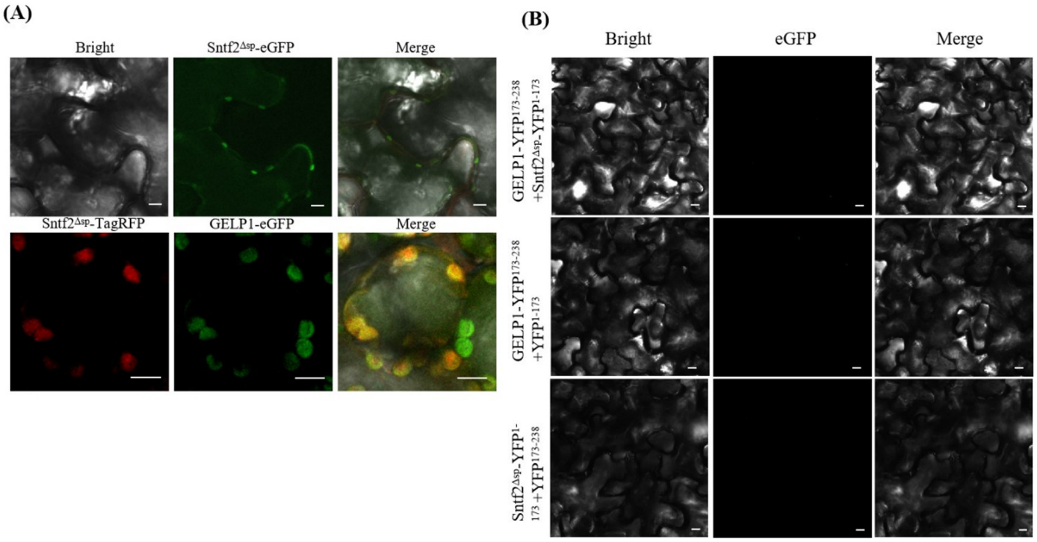

Figure S2. Detection of the GELP1–Sntf2 interaction using co-localization and bimolecular fluorescence complementation assays. Bar = 10 μm.

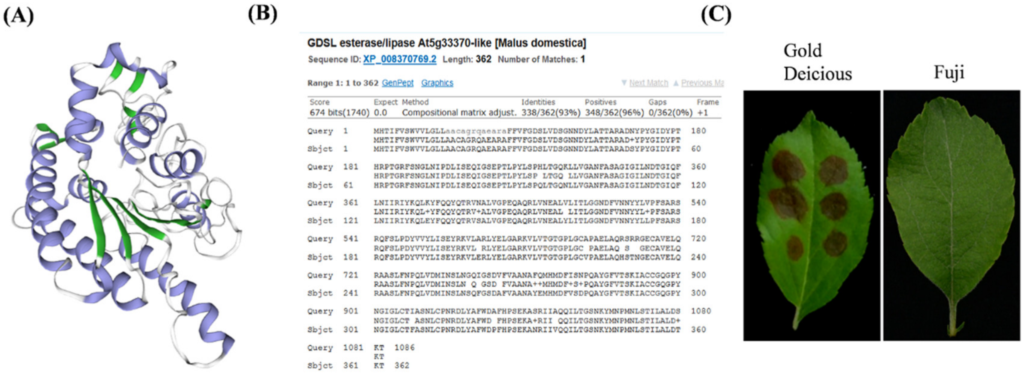

Figure S3. Identification the structure and sequence alignment of *GELP1* and *GELP2*. (**A**) Prediction of the three dimensions structure of *GELP1* using SWISS-MODEL (https://swissmodel.expasy.org/). (**B**) Sequence alignment of *GELP1* and *GELP2* from NCBI blastP, and alignment data was generated using the DNAMAN software (version 6). (**C**) The pathogenicity of *C. gloeosporioides* on Gold Delicious and Fuji leaves.
