# Supplementary material for: GDSL Esterase/Lipase GELP1 Involved in the Defense of Apple Leaves against Colletotrichum gloeosporioides Infection
Source: Int J Mol Sci. 2023 Jun 19;24(12):10343. doi: 10.3390/ijms241210343 (PMC10299637; doi:10.3390/ijms241210343)
Supplement: Supplementary file 1 [file ijms-24-10343-s001.zip › Tables S1 and S2.pdf]

**Table S1.** *Cis*-elements analysis of *GELP1* promoter

| *Cis*-elements | Sequence | Position | Function |
|---|---|---|---|
| TC-rich repeats | ATTCTCTAAC | 1095 | cis-acting element involved in defense and stress responsiveness |
| Box 4 | ATTAAT | 376 | part of a conserved DNA module involved in light responsiveness |
| ABRE | ACGTG | 408 | cis-acting element involved in the abscisic acid responsiveness |
| ATCT-motif | LOC114824837 | 2007 | part of a conserved DNA module involved in light responsiveness |
| CGTCA-motif | CGTCA | 829 | cis-acting regulatory element involved in the MeJA-responsiveness |
| G-Box | CACGTT | 408 | cis-acting regulatory element involved in light responsiveness |
| G-box | TAACACGTAG | 866 | cis-acting regulatory element involved in light responsiveness |
| GATA-motif | AAGGATAAGG | 546 | part of a light responsive element |
| HD-Zip 1 | CAAT(A/T)ATTG | 750 | element involved in differentiation of the palisade mesophyll cells |
| TCA-element | CCATCTTTTT | 1537 | cis-acting element involved in salicylic acid responsiveness |
| TGA-element | AACGAC | 66 | auxin-responsive element |
| Box 4 | ATTAAT | 658 | part of a conserved DNA module involved in light responsiveness |

**Table S2.** *Cis*-elements analysis of *GELP2* promoter

| *Cis*-elements | Sequence | Position | Function |
|---|---|---|---|
| 3-AF1 binding site | TAAGAGAGGAA | 921 | light responsive element |
| ABRE | ACGTG | 872 | cis-acting element involved in the abscisic acid responsiveness |
| ABRE | ACGTG | 782 | cis-acting element involved in the abscisic acid responsiveness |
| ARE | AAACCA | 1664 | cis-acting regulatory element essential for the anaerobic induction |
| ARE | AAACCA | 649 | cis-acting regulatory element essential for the anaerobic induction |
| ATCT-motif | AATCTAATCC | 129 | part of a conserved DNA module |

| | | | |
|---|---|---|---|
| | | | involved in light responsiveness |
| ATCT-motif | AATCTAATCC | 1920 | part of a conserved DNA module involved in light responsiveness |
| CGTCA-motif | CGTCA | 1552 | cis-acting regulatory element involved in the MeJA-responsiveness |
| G-box | TACGTG | 871 | cis-acting regulatory element involved in light responsiveness |
| GATA-motif | GATAGGG | 439 | part of a light responsive element |
| GC-motif | CCCCCG | 419 | enhancer-like element involved in anoxic specific inducibility |
| MRE | AACCTAA | 1669 | MYB binding site involved in light responsiveness |
| MRE | AACCTAA | 1743 | MYB binding site involved in light responsiveness |
| TCT-motif | TCTTAC | 868 | part of a light responsive element |
